# Supplementary material for: Increased Tolerance of Massion’s pine to Multiple-Toxic-Metal Stress Mediated by Ectomycorrhizal Fungi
Source: Plants (Basel). 2023 Sep 5;12(18):3179. doi: 10.3390/plants12183179 (PMC10535352; doi:10.3390/plants12183179)
Supplement: Supplementary file 1 [file plants-12-03179-s001.zip › plants-2538787-supplementary.pdf]

1 Supplementary Figures and Tables

1.1 Supplementary Tables

Supplemental Table S1. Statistical table for evaluation of sequencing data of different samples.

Samples	Read Number	Base Number	GC Content(%)	%≥Q30
NM-1	22710849	6792499676	47	96.41
NM-2	22340133	6671117474	46.48	95.88
NM-3	22139007	6619599198	47.32	95.46
CG1-1	16881219	5039195896	46.51	95.44
CG1-2	14223585	4250836346	46.5	94.92
CG1-3	21380484	6396300174	46.07	95.78
CG2-1	19419383	5812151538	45.46	95.93
CG2-2	26228489	7847909142	46.27	94.06
CG2-3	21386178	6401791232	46.09	95.87

Non-inoculated (NM) and inoculated (CG1, CG2); Read Number refers to total number of pair-end reads in clean data; Base Number refers to the total number of bases in clean data; GC content refers to clean data GC content, that is the percentage of G and C bases in total bases in clean data; Q30% refers to the percentage of bases with a mass value of 30 or greater in clean data.

Supplemental Table S2. Statistical table of assembly results.

Length Range	Transcript	Unigene
300-500	15,316(20.43%)	11,492(30.11%)
500-1000	9,094(12.13%)	5,041(13.21%)
1000-2000	18,639(24.86%)	9,109(23.87%)
>2000	31,928(42.58%)	12,523(32.81%)
Total Number	74977	38165
Total Length	140,479,335	60143803
N50 Length	2515	2354
Mean Length	1873.63	1575.89

Length Range refers to different length intervals of Unigene; The number in the table represents the number of Unigene in the corresponding interval, and the percentage in parentheses refers to the proportion of Unigene in the corresponding length interval; Total Number refers to the total number of assembled Unigenes; Total length refers to the total length of the assembled Unigene; N50 Length refers to the length of N50 of Unigene; Mean Length refers to the average length of Unigene.

Supplemental Table S3. Sequences of primers used for RT-qPCR analysis in this paper.

Primer	Sequence(5'-3')
TRINITY_DN81698_c0_g1-F	AGCAGCACGGTTCAACAAAC
TRINITY_DN81698_c0_g1-R	AGCTTCCAATGTGCTGCAAG
TRINITY_DN156398_c0_g1-F	AGCTGCGGCAAAATTCAAGG
TRINITY_DN156398_c0_g1-R	TGACGCCATCAATCGCTTTC
TRINITY_DN22597_c0_g2-F	GCTACAGCTTTCCTGGGTTATG
TRINITY_DN22597_c0_g2-R	TCCAACCCAAGGAATTGCAC
TRINITY_DN40863_c0_g1-F	TGGGCTGGTTCTTGAATTGC
TRINITY_DN40863_c0_g1-R	TTCACTGCGTCAATGTTCCG
TRINITY_DN55799_c0_g1-F	AGCTTGACGCGGATTTCTTC
TRINITY_DN55799_c0_g1-R	AAGCCAAGCAAACCATGACG
TRINITY_DN4024_c0_g1-F	AACCGTTTTGGCGCCATTAG
TRINITY_DN4024_c0_g1-R	TTGGGCGCAAGTTCCTTTTC
TRINITY_DN74597_c0_g1-F	ACGCGCTCTTCTTGCAATTG
TRINITY_DN74597_c0_g1-R	TTTGGAACGGAGCATTGACG
TRINITY_DN17585_c0_g1-F	ATTGCCCCGAAAGAAATCCG
TRINITY_DN17585_c0_g1-R	TTTTTCGCACCACCATCGAC
Aquaporin protein gene(AQP)-F	GGATTTGGTCGTATTGGGAGG
Aquaporin protein gene(AQP)-R	TTTGGCATCAATGAAAGGGTC

1.2 Supplementary Figures

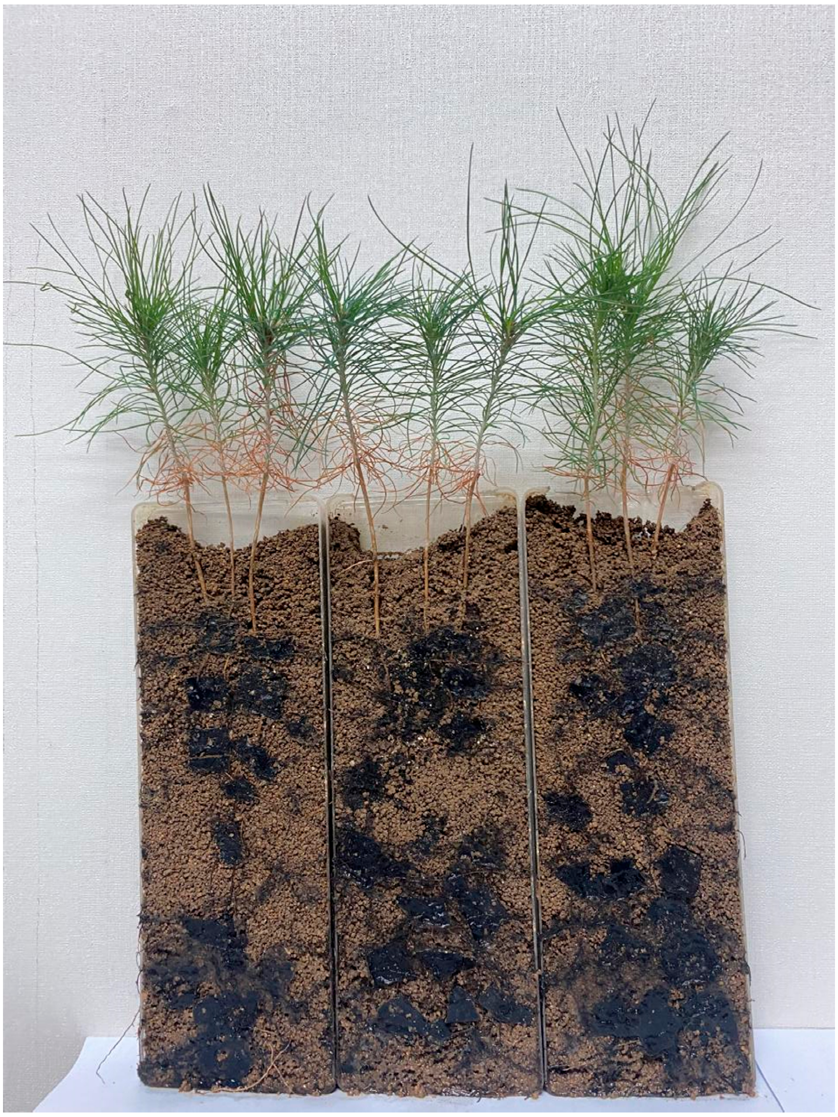

Figure S1. *Massion's pine* inoculated with *Cenococcum geophilum* (CG)

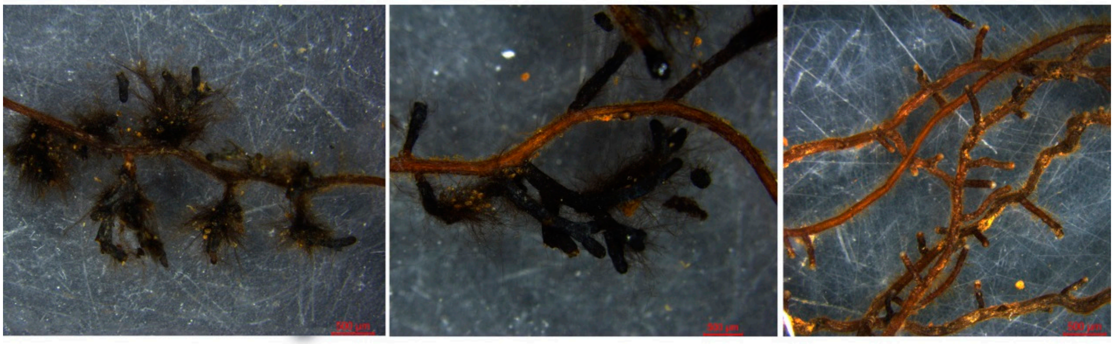

CG1 CG2 NM

Figure S2. *Massion's pine* inoculated with *Cenococcum geophilum* (CG1, CG2) and non-inoculated plants (NM).
